# A rare case of granular cell tumor of gall bladder

**DOI:** 10.1093/jscr/rjaf071

**Published:** 2025-02-19

**Authors:** Sunil Dhakal, Sapana Bhandari, Avinam H Kandangwa

**Affiliations:** Gastrointestinal Surgical Unit, B.P. Koirala Memorial Cancer Hospital, Bharatpur 7, Chitwan 44200, Bagmati Province, Nepal; Gastrointestinal Surgical Unit, B.P. Koirala Memorial Cancer Hospital, Bharatpur 7, Chitwan 44200, Bagmati Province, Nepal; Gastrointestinal Surgical Unit, B.P. Koirala Memorial Cancer Hospital, Bharatpur 7, Chitwan 44200, Bagmati Province, Nepal

**Keywords:** granular cell tumor, gall bladder, CD 68, S 100

## Abstract

Granular cell tumor is common over skin and subcutaneous and oral cavity. However, lesion is rare in biliary tract and ever more uncommon in gall bladder. These lesions are often misdiagnosed until proven by histology and immunohistochemistry where tumor is positive for CD 68, S 100 which points them toward neural origin of tumor. We report a case of 69-year-old male referred with a suspected gall bladder mass in Computed tomographic scan of abdomen. Laparotomy was done through Kocher’s incision and extended cholecystectomy was performed. Histopathology and immunohistochemical study helped in determining gall bladder lesion to be granular cell tumor. Therefore, granular cell tumor can be considered a possible differential diagnosis of all the gall bladder lesion suspected of cholecystitis and carcinoma. Margin negative excision of the tumor is a curative approach for the patient.

## Introduction

Granular cell tumor is a very uncommon benign tumor, moreover, being a gall bladder (GB) origin, a rarity [1]. Malignant transformation of the tumor is rare. Most common location of the tumor is oral cavity, skin, and subcutaneous tissue however due to difficult accessibility identification of the tumor at GB and bile duct is challenging [2]. Patient commonly present with feature of acute or chronic cholecystitis which further causes diagnostic dilemma [2].

## Case report

A 69-year-old male belonging to hilly region of Nepal was referred to BPKMCH with a suspected GB carcinoma for which triphasic computed tomographic scan of thorax abdomen and pelvis was done, which had revealed GB fundus mass with surrounding liver lesion. Ultrasonographic FNAC was done from liver lesion and GB mass, which suspected it of malignancy. Patient was received at surgical gastro oncology unit with above mentioned reports. On presentation patient had pain over epigastrium. His bladder and bowel habit were normal including his weight. His past medical history was unremarkable. Thorough physical examination of the abdomen revealed hard nontender mass palpable in the right upper quadrant of the abdomen.

**Figure 1:**
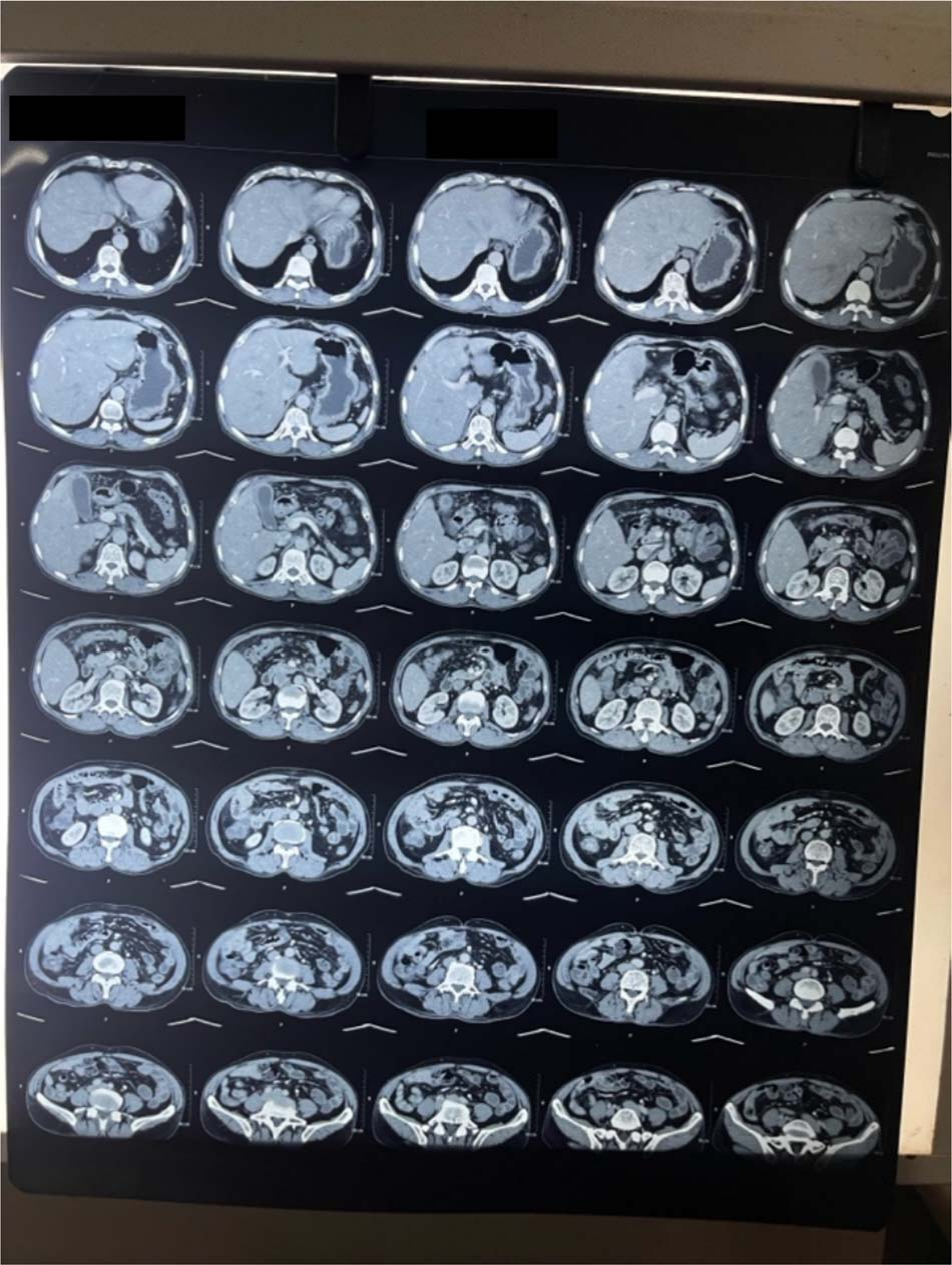
CT abdomen and pelvis transverse cut shows thickened GB wall.

**Figure 2:**
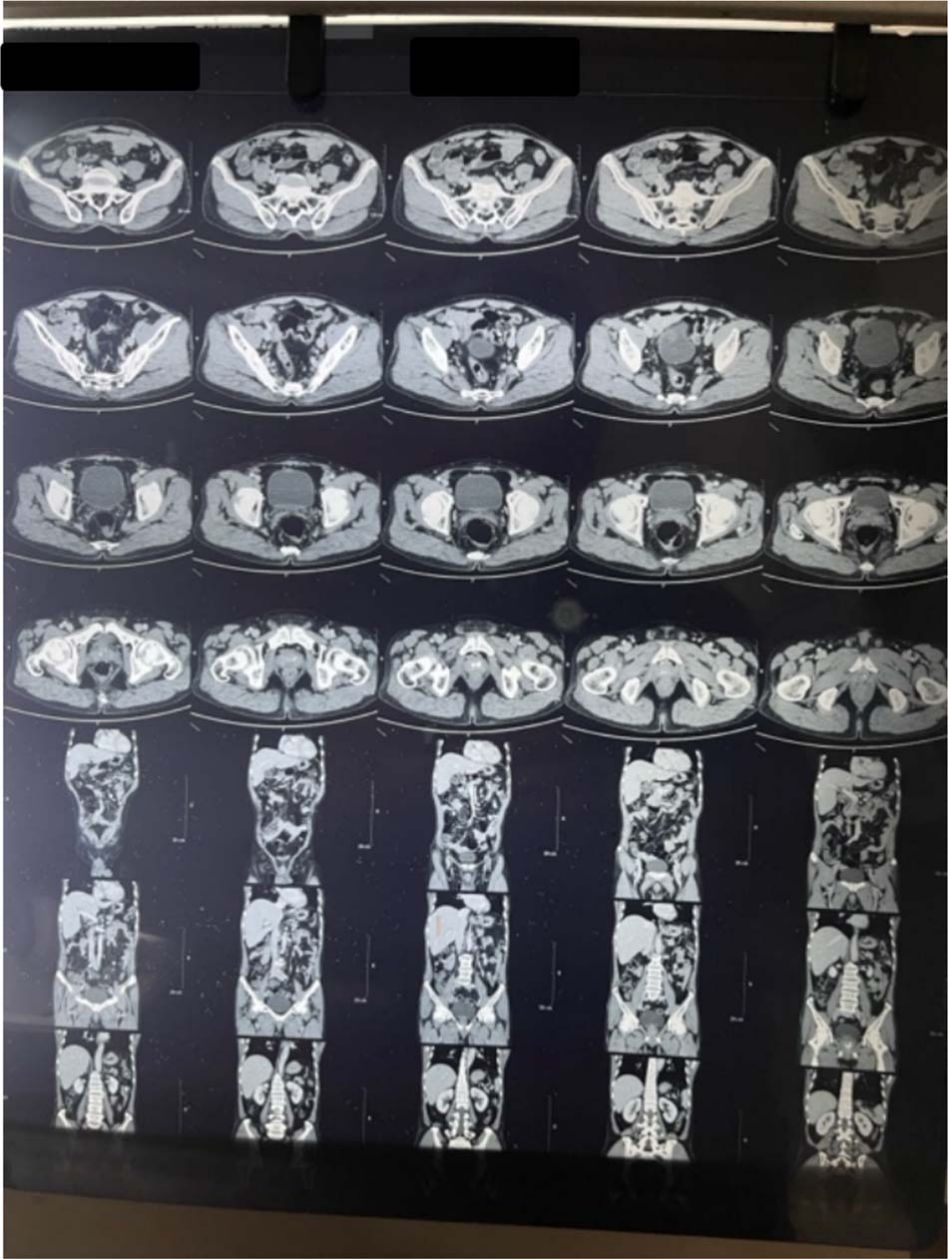
CT coronal cut abdomen and pelvis shows thickened wall.

Laboratory analysis showed hemoglobin of 15.0 g/dl, total leucocyte count of 9840/cumm, total bilirubin of 2.7 mg/dl, direct bilirubin of 1.7 mg/dl, ALP of 176.3 U/L, AST, and ALT level of 303 IU/L and 512.5 IU/L, respectively. HBsAg, HCV, and HIV serology was non- reactive. Tumor marker (CA19.9) was 998 U/ml. Abdominal ultrasonography revealed thickened GB fundic wall with cholelithiasis. Computed tomography of the abdomen revealed hypodense enhancing lesion of 14 × 28 × 17 mm noted in liver surrounding fundus of GB with peri portal and upper retroperitoneal lymphadenopathy with cholelithiasis and choledocholithiasis (Figs 1 and 2).

Patient was admitted. Kocher’s incision was given for a laparotomy. Irregular mass of GB fundus with infiltration to liver noted along with multiple enlarged lymph nodes along level 12a, 12p, 12 c. No evidence of omental, pelvis, mesenteric, and hepatic metastasis found. Extended cholecystectomy with 2 cm of liver margin was excised. Common bile duct (CBD) stones were retrieved through open CBD exploration. Post-operative period was uneventful.

**Figure 3:**
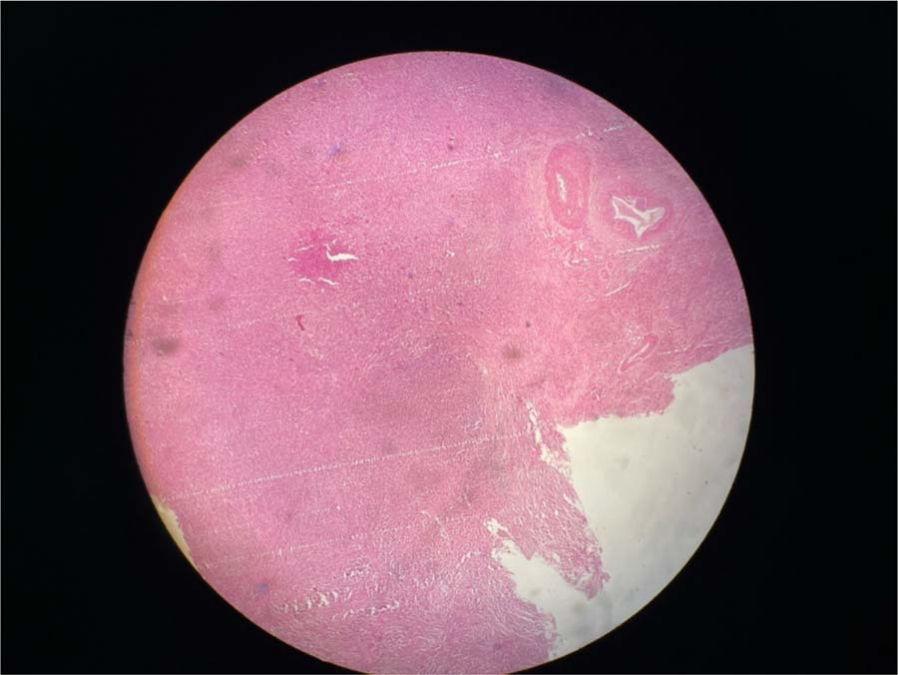
Microscopic picture shows polygonal eosinophilic cells arranged in clusters and diffusely infiltrates the surrounding cell.

**Figure 4:**
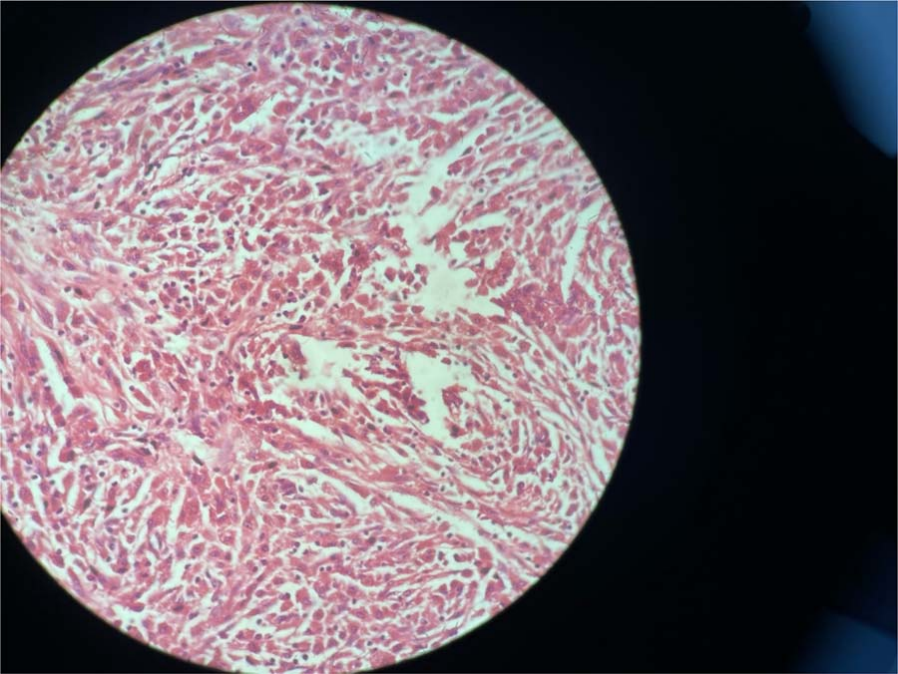
High resolution image shows polygonal cells with granular cytoplasm with vesicular small centrally located nucleus.

**Figure 5:**
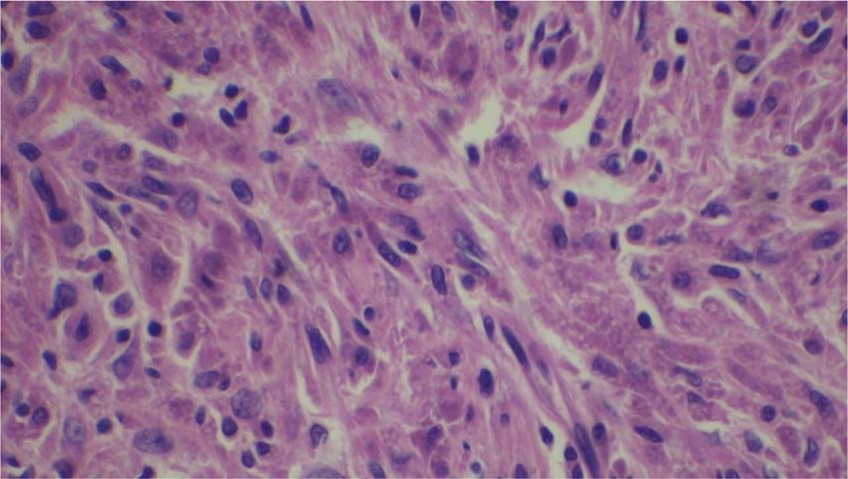
H and E stain.

**Figure 6:**
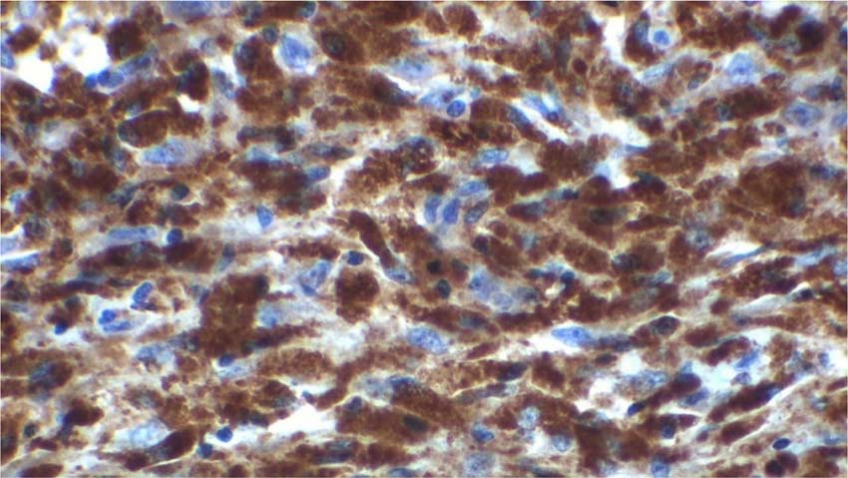
CD 68 positivity (KP 1).

Gross examination revealed mass along GB 5 × 2 × 1 cm and attached 5 × 4 × 2 cm liver. Cut section of GB mucosa is velvety and fundus is thickened measuring 1.2 cm. Histopathology report revealed polygonal eosinophilic cells with granular cytoplasm strongly positive for periodic acid-Schiff (PAS) (Figs 3 and 4). These cells also contain vesicular small centrally located nucleus which appears in clusters or sheets and infiltrated diffusely within the surrounding structures suggesting of Granular cell tumor. All the margins were negative for tumor. Liver was unremarkable, so were the regional lymph nodes. Immuno-histochemistry confirmed CD 68 positivity and negative for SOX10, CK, SMA, desmin, Ki-67 favoring the neural origin of the tumor further enhancing the diagnosis (Figs 5 and 6).

## Discussion

Granular cell tumor was first described in 1926 by Abrikossoff as myoblastic myomata in the skeletal muscle of tongue. Immunohistochemical S100, CD 68, protein gene product 9.5 and inhibin alpha protein positivity points toward neural origin of the tumor, likely being Schwann-like mesenchymal cell [3, 4]. Granular cell tumor can occur at any age but more prevalent in people in their 40s and 50s of age. There is slight predominance among African American females [2].

Granular cell tumor is common in tongue, oropharynx, GIT, skin, and subcutaneous tissue, breast, and respiratory tract. Biliary tract granular cell tumor is of rare presentation. Among biliary tract granular cell tumor GB occurrence is <2.3% making it a rarity [3, 5, 6]. Granular cell tumor of GB presents with acute on chronic cholecystitis or chronic cholecystitis and rarely with thickened GB wall as a suspect of carcinoma [2, 3]. Unless proven by histology granular cell tumor is difficult to diagnose preoperatively in GB. Based on radiological findings patient undergo complex surgical procedures like extended cholecystectomy for GB lesion and up to Whipple’s procedure for biliary tract lesions [2, 7].

Gall bladder and biliary tract lesion are mostly benign hence are cured with complete surgical excision. Tumor prognosis therefore is good after complete excision. Till date no malignant granular cell tumor of biliary tract have been reported however two cases of recurrence have been known due to incomplete removal of tumor [2, 8].

Microscopically these tumors contain granular eosinophilic cytoplasm with vesicular nucleus where granules are positive for periodic-acid Schiff’ staining. They appear in cluster or sheets. They infiltrate surrounding tissue diffusely separated by thin connective tissue. Mitoses are rare. No necrosis is present. Reactive atypia of the overlying epithelium and metaplastic pyloric gland may mimic carcinoma [7, 9, 10]

Granular cell tumor is rare among Asian populations. Disease is more predominant among young black women. GB granular cell tumor is very unusual and rare [8].

## Conclusion

Granular cell tumor of GB is a rare benign tumor among Asian population. They present with the feature of acute on chronic cholecystitis or chronic cholecystitis and computed tomographic picture of GB carcinoma leading to extensive operative procedures such as extended cholecystectomy as in our case. The lesion is often misdiagnosed clinically. However timely intervention and local excision of mass with adequate margins is curative for the patient.
